# A dataset of soil-sweep tool interaction measurements on an agricultural field including cone penetration resistance, draft force, and surface profiles under varying soil moisture conditions

**DOI:** 10.1016/j.dib.2026.113247

**Published:** 2026-09-04

**Authors:** Dániel Nagy, Kornél Tamás

**Affiliations:** a Department of Hydrodynamic Systems, Faculty of Mechanical Engineering, Budapest University of Technology and Economics, Műegyetem rkp. 3, H-1111, Budapest, Hungary; b Department of Machine and Product Design, Faculty of Mechanical Engineering, Budapest University of Technology and Economics, Műegyetem rkp. 3, H-1111, Budapest, Hungary

**Keywords:** Tillage, Cultivator sweep, Agricultural field measurements, Clay loam soil, Laser profilography, Discrete element method, Validation data

## Abstract

This dataset contains field measurements of soil-sweep tool interactions in clay loam soil acquired at the research field of the Institute of Technology of the Hungarian University of Agriculture and Life Sciences in Gödöllő, Hungary. Measurements were conducted during three field campaigns representing distinct soil moisture conditions over the course of one year. The objective of the measurement campaign was to document soil conditions, tillage parameters, and soil response using a consistent experimental methodology at the same agricultural field. Cone Penetration Tests (CPT) were performed at multiple locations within each campaign using an Eijkelkamp 06.15.SA penetrologger, while soil moisture was measured at the same locations. Tillage experiments were carried out using three sweep tools with different working widths operated at three target working speeds. During each tillage run, draft force and speed were continuously recorded using a custom instrumented tractor. Surface profiles were measured before and after tillage using a laser profilograph for the dry and medium-moisture soil conditions. The repository is organized according to soil moisture conditions and includes raw measurement files and accompanying JSON metadata. In addition, scanned 3D models of the investigated sweep tools are provided in STL format. The dataset can be reused for studies of soil moisture effects on tillage performance, development and validation of discrete element method (DEM) and finite element method (FEM) models, and for benchmarking soil-tool interaction simulations.

Specifications Table

**Table:** 

Subject	Earth & Environmental Sciences
Specific subject area	***Agriculture, Soil Tillage, Discrete Element Method***
Type of data	Table (Raw, Processed)
Data collection	All data were acquired during field measurements. Cone penetration tests were carried out using an Eijkelkamp 06.15.SA penetrologger equipped with a 1cm^2^, 60° cone and with a 2cm/s penetration velocity. Draft force during tillage was measured using a KYOWA KFC-10-C1–11 strain-gauge, while the working speed was measured with an OMRON E2A sensor attached to the wheel. Both were recorded with 50 Hz sampling rate by a QuantumX MX840 B data collector. Surface profiles were measured before and after each run in the dry and medium soils using a laser profilograph (OPTRON ODS 750, ASM WS19KT-15,000). The recorded soil profiles were then normalized to set the coordinate center to the location of the tool.
Data source location	Data were collected at the research field of Institute of Technology of the Hungarian University of Agriculture and Life Sciences, Hungary. City: Gödöllő Country: Hungary Latitude and Longitude: 47°35′29.2″N 19°20′04.7″E
Data accessibility	Repository name: Agricultural field measurements of soil-sweep tool interactions over one year Data identification number: 10.5281/zenodo.21,343,213 Direct URL to data: https://zenodo.org/records/21343213 License: CC-BY-4.0 Instructions for accessing these data: The data can be freely downloaded. Following download, the archive must be extracted.
Related research article	*None*

## 1 Value of the Data

• The dataset provides agricultural field measurements in clay loam soil for investigating the influence of soil moisture, tool geometry, and operating speed on soil-sweep tool interactions. By combining soil moisture measurements, cone penetration resistance, tillage draft forces, and surface profile measurements, the dataset enables analysis of how varying soil conditions affect both soil behavior and tillage performance.
• The acquisition of such field measurements requires specialized agricultural machinery, dedicated sensors, access to an agricultural field that remains undisturbed between measurement campaigns, and substantial experimental effort. The measurement campaigns required a tractor equipped with custom force and speed recording systems, multiple field preparations, and numerous on-site measurements, including cone penetration testing, tillage experiments, surface profile acquisition, and soil characterization.
• Data from three measurement campaigns are available, each with distinct soil moisture conditions within the same agricultural field covering a full year. Cone penetration tests were conducted at 15 locations under dry soil conditions, 3 locations under medium moisture conditions and 10 locations under wet soil conditions. Nine tillage runs were conducted during each of the dry and medium moisture campaigns, covering the combinations of three sweep tools and three working speeds. During the wet-soil campaign, one parameter combination was repeated, resulting in a total of 10 tillage runs. Draft force and velocity were recorded continuously during all tillage runs. Surface profiles were measured before and after each tillage operation for the dry and medium moisture soil conditions.
• The dataset serves as a benchmark resource for the development and validation of numerical soil-sweep tool interaction models. Scanned 3D models of the used soil sweep tools are available in the dataset in STL format. The combination of soil characterization data, operational parameters, and measured responses supports the calibration and validation of discrete element method (DEM) models, finite element method (FEM) models, and other predictive approaches used in agricultural engineering and terramechanics.
• The dataset also supports the development of data-driven methods for predicting tillage performance and optimizing field operations. Researchers can use the data to establish relationships between soil properties, operating conditions, draft force requirements, and soil surface modifications. All measurements were performed on the same agricultural field over a one-year period, reducing variability associated with differences in soil type.

## 2 Background

Soil-tool interaction is strongly influenced by soil mechanical properties, operating conditions, and tool geometry, with soil moisture being an important factor affecting soil behavior [[1], [2]]. Changes in moisture content can substantially alter soil strength, deformation characteristics, and tillage performance, making field measurements under different moisture conditions particularly valuable. However, comprehensive experimental datasets acquired under realistic agricultural field conditions are relatively scarce, as many studies rely on controlled laboratory experiments [[3], [4]]. The present dataset was compiled during a field measurement campaign conducted under three distinct soil moisture conditions within the same agricultural field. The primary motivation was to obtain a consistent set of measurements for the development, calibration, and validation of soil-sweep tool interaction models, including discrete element method (DEM) simulations [[5], [6], [7], [8]].

## 3 Data Description

The dataset is distributed within the Measurements directory and is organized according to the soil moisture condition during each measurement campaign. The repository contains a top-level metadata file describing the measurement campaigns and the soil, including the measurement date, soil moisture condition, number of measurements available for each campaign and the properties of the soil. The summary of the measurement campaigns is also given in Table 1. The date of the measurement and the average soil moisture content is specified, then the number of available data series is given for each type of measurement. The Measurements directory contains three subfolders: Dry, Medium, and Wet. Each moisture-condition folder contains two subdirectories: CPT and Tillage.

**Table 1:** Summary of measurement campaigns.

Date	Soil moisture content (volumetric)	CPT tests	Tillage runs	Surface profile measurements
August 27, 2018	6.80% (dry)	15	9	9
December 5, 2018	22.10% (wet)	10	10	0
April 12, 2019	13.33% (medium)	3	9	8

**Fig. 1:**
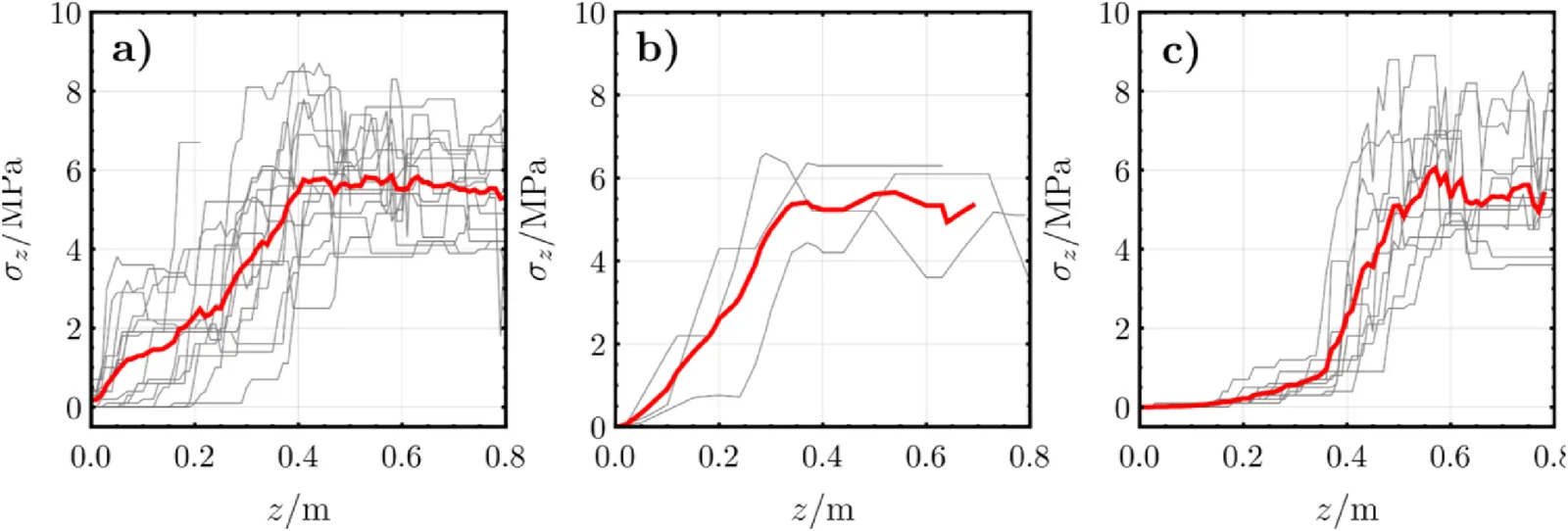
Cone penetration resistance *σ_z_* as a function of penetration depths *z* for (a) dry soil (6.8% v/v moisture content); (b) medium soil (13.33% v/v moisture content) and (c) wet soil (22.1% v/v moisture content). The gray curves show individual measurements while the red curve is the average of all measurements.

The CPT folders contain all cone penetration test (CPT) measurements acquired during the corresponding measurement campaign. Each measurement is provided as an individual CSV file containing penetration depth (m) and cone penetration resistance (MPa). An accompanying metadata file provides the measurement identifier, volumetric soil moisture content, and GPS coordinates of the measurement location. Fig. 1 illustrates the CPT measurements obtained under dry, medium and wet soil conditions. Individual penetration resistance profiles are shown in gray, while the average profile corresponding to each moisture condition is shown in red.

**Fig. 2:**
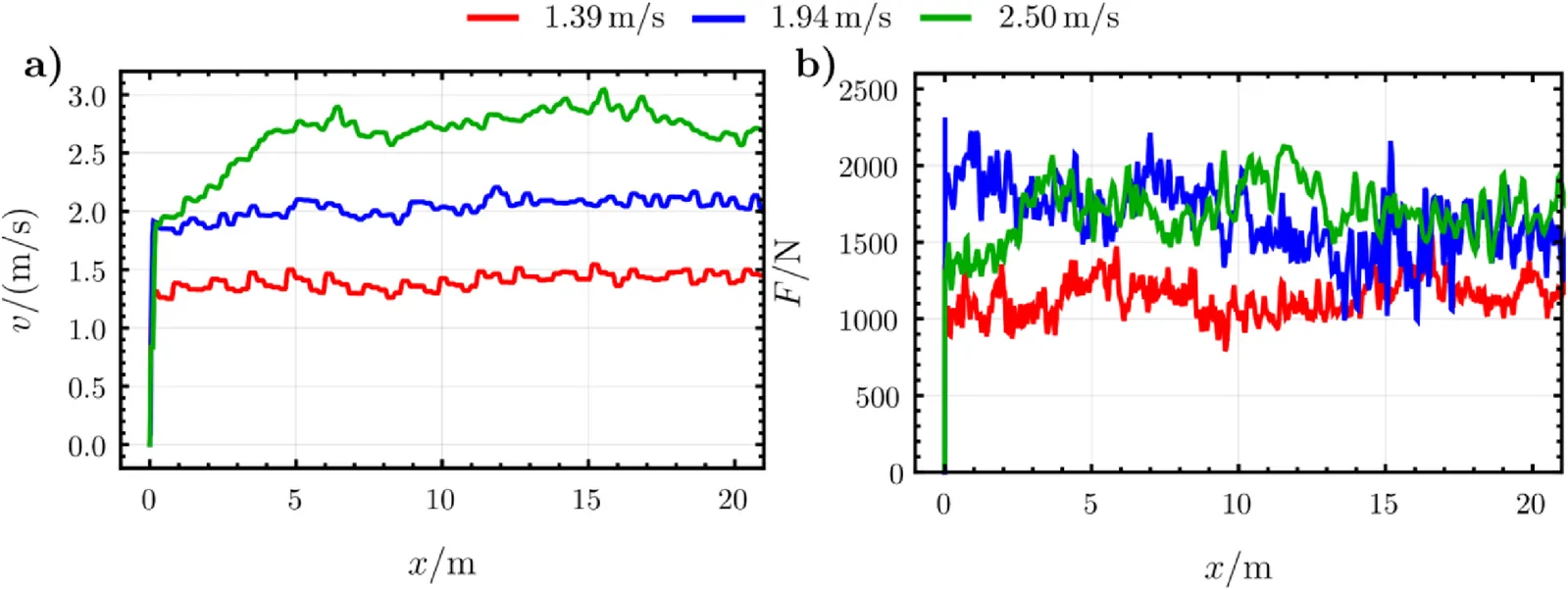
Representative tillage measurements with the smallest tillage tool under dry soil conditions. (a) Recorded speed *v* and (b) recorded draft force *F* as a function of displacement *x*. The legend indicates the average velocity calculated over the displayed interval.

The Tillage folders contain all draft-force measurement data obtained during the tillage experiments. Each tillage run is stored as an individual CSV file containing the recorded time (s), displacement (m), velocity (m/s), and draft force (N). A metadata file accompanies the measurements and specifies the tillage tool, nominal operating velocity, measurement identifier, and measurement conditions. For each run, the metadata file additionally contains the displacement interval over which approximately constant operating speed was maintained. Within this interval, the mean and standard deviation of both velocity and draft force are reported. Fig. 2 presents representative measurements obtained using the smallest tillage tool under dry soil conditions at various working speeds. The figure shows the recorded velocity and draft force over the first 20 m of displacement for three measurement runs, for the various nominal speed settings according to the legend.

**Fig. 3:**
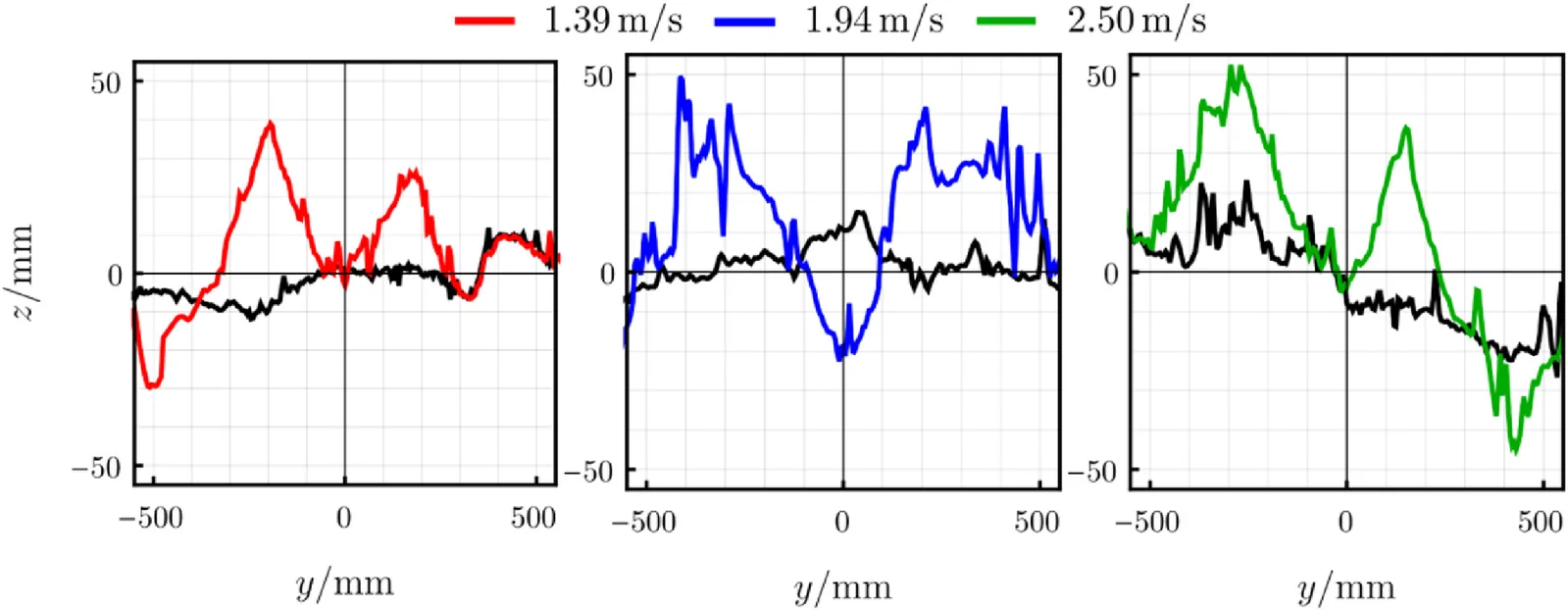
Surface profiles before the tillage (black line) and following the tillage measurement (colored, see legend). Measurements are carried out with the small sweep tool in dry soil. The center of the tillage tool is located at *y=0*.

Surface profile data are also stored within the corresponding Tillage folders. For each run, the dataset contains post-processed results of measurements of the soil surface acquired before and after tillage. The CSV files contain the horizontal coordinate location (*y* in mm) and the measured surface elevation (*z* in mm). Fig. 3 shows representative surface profiles measured before and after soil sweep, for the same three measurement runs presented in Fig. 2.

Each measurement campaign contains metadata files in JSON format describing the corresponding measurements. In the CPT folders, the metadata first specifies the cone geometry and operating conditions, including the cone apex angle, projected base area, and penetration velocity. The units of all recorded quantities are then provided. For each measurement, the metadata includes the path to the corresponding CSV file, the measured volumetric soil moisture content, GPS coordinates, and the maximum penetration depth. In the Tillage folders, the metadata file first specifies the sweep tool dimensions and the units of all recorded quantities. For each measurement, the metadata includes the paths to the corresponding tillage and surface profile data files, together with the nominal operating velocity set on the tractor. The metadata further defines a quasi-steady operating interval, specified both in terms of time and tractor displacement. Within this interval, the velocity and draft force are expected to fluctuate around approximately constant values. For this interval, the mean and standard deviation of both tractor velocity and draft force are reported.

## 4 Experimental Design, Materials and Methods

### 4.1 Experimental site

All field measurements were conducted at the same agricultural field near Gödöllő, Hungary, during three measurement campaigns representing distinct soil moisture conditions. The field campaign was designed to investigate soil-sweep tool interactions under varying soil moisture, tool geometry, and operating speed with consistent measurement methodology. The same measurement protocol was repeated during each campaign, including cone penetration tests, tillage measurements, and surface profile measurements. Sample locations were recorded using GPS coordinates.

### 4.2 Characterization of the soil

Soil samples collected from the experimental field were analyzed in the geotechnical laboratory of Budapest University of Technology and Economics before the field campaigns. Laboratory analyses included particle size distribution, density determination, and consistency measurements. The particle size distribution analysis showed that the investigated soil consisted of approximately 35.13% sand, 36.33% silt, 28.10% clay, and 0.44% gravel by mass, indicating a fine-grained agricultural soil with a substantial silt and clay fraction that can be classified as a clay loam. The measured particle density was 2700 kg/m^3^. Consistency testing yielded a liquid limit of 45.1%, a plastic limit of 18.4%, and a plasticity index of 26.7%.

### 4.3 Cone penetration tests

Cone Penetration Tests (CPT) were conducted before the tillage experiments during each measurement campaign to characterize the mechanical state of the soil. The measurements were performed at multiple locations distributed across the experimental field. For each measurement location, the geographical coordinates were recorded. The CPT measurements were carried out using a hand-operated Eijkelkamp 06.15.SA penetrologger (serial number: 34,445,101). The device consisted of a rod equipped with a conical tip having an apex angle of 60° and a projected base area of 1 cm². During each test, the cone was inserted vertically into the soil at a nearly constant penetration rate of 2 cm/s until a depth of approximately 0.8 m was reached. The penetration resistance was continuously recorded as a function of depth. The measured force acting on the cone was converted by the instrument into cone penetration resistance values. The resulting data were stored as depth-resistance profiles for each measurement location. Multiple measurements were performed within each moisture condition to account for spatial variability within the field. In addition to penetration resistance, soil moisture measurements were also recorded at each CPT location using a Thetaprobe ML3 (1% v/v accuracy).

### 4.4 Draft-force measurements during tillage

**Fig. 4:**
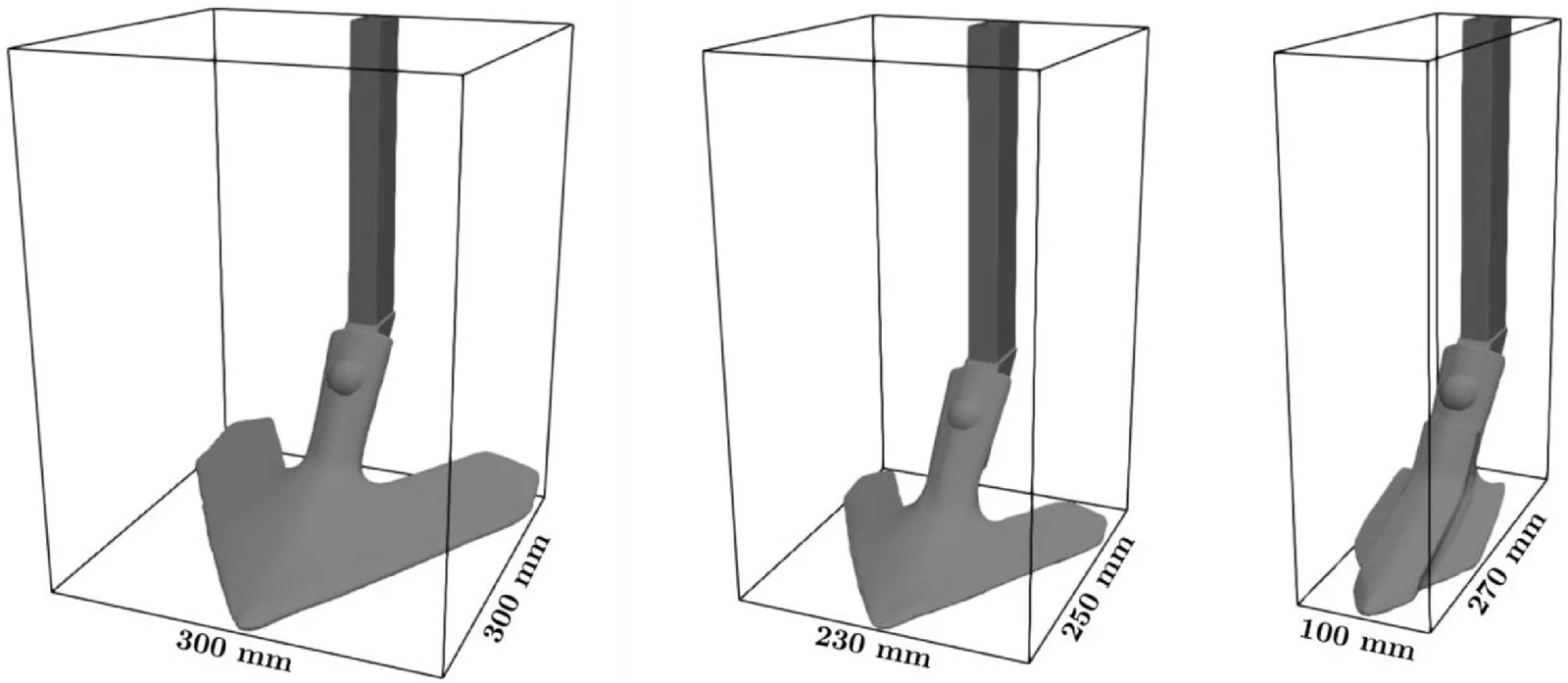
The 3D scan of sweep tools used during measurements.

Tillage experiments were performed during each measurement campaign using three sweep tools with different working widths. The investigated tools had approximate widths of 300 mm, 230 mm, and 100 mm and were mounted individually on a tractor. The 3D scans of these tools are depicted in Fig. 4, ordered from large to small. A nominal tillage depth of 150 mm was set and verified before each run. Surface profiles measured on the prepared soil showed a standard deviation of 7.7 mm in surface elevation. Although these profiles were not measured along the paths traversed by the tools, they characterize the soil preparation method applied throughout the experimental area. Consequently, a similar magnitude of variation in the actual tillage depth is expected. For each soil moisture condition, every tool was tested at three target working speeds, resulting in nine tillage runs per campaign (in wet soil, the small tool with the lowest nominal speed was tested twice). Each run was carried out over a field section approximately 60–100 m long. After completion of a measurement run, the soil surface was manually prepared again before the next experiment was initiated.

**Fig. 5:**
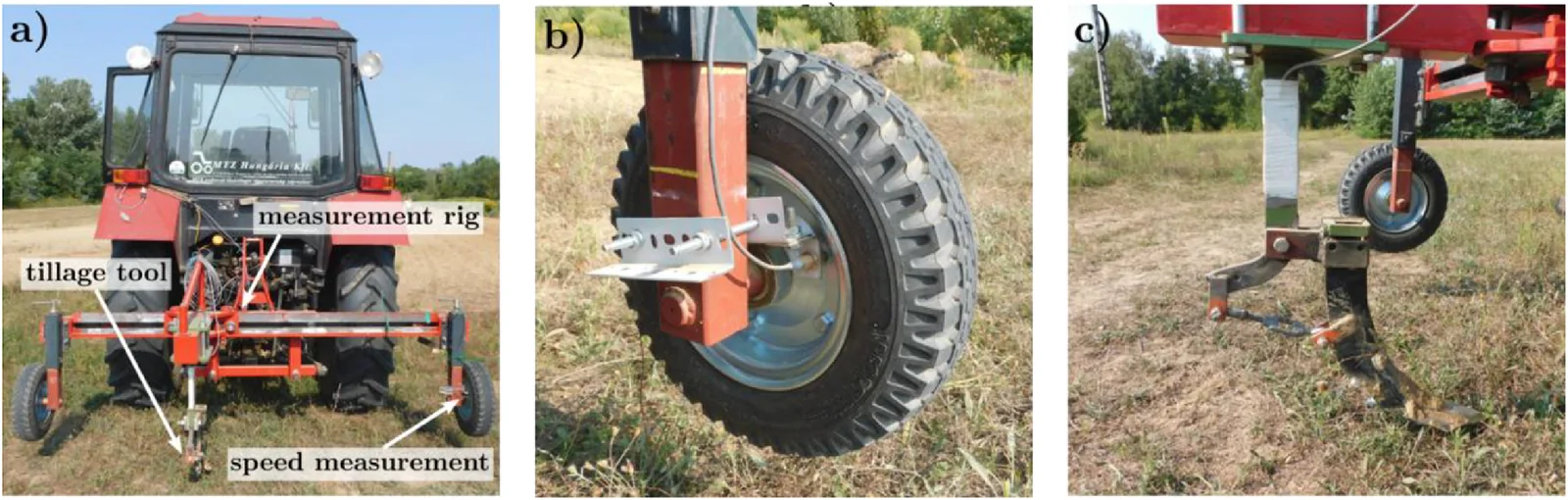
(a) The measurement rig fixed to a tractor, with the sweep tool and rotational speed measurement device. (b) The mounting of the rotation speed measurement device. (c) The mounting of the sweep tool.

Fig. 5 depicts the custom measurement rig that was attached to the tractor. The draft force was measured by KYOWA KFC-10-C1–11 strain gauges connected to the shanks. After installation, the strain gauges were calibrated using a custom calibration setup with 8 different loads. Each instrumented tool was mounted in the same configuration as during the field measurements but rotated by 90°. Based on the differences between the applied forces and the forces predicted using the calibration relationship, a mean absolute error of 2.5 N was obtained.

The measurement signals were recorded using a QuantumX MX840 B device and the catmanEasy data acquisition software, with a sampling frequency of 50 Hz. During the runs, the draft force acting on the tool and the tractor velocity were recorded continuously as a function of time. Working speed was determined from the wheel rotation using an OMRON E2A proximity sensor as depicted in Fig. 5b. During the wet-soil campaign, wheel slip affected the operation of the instrumented wheel in all 10 measurement runs. Therefore, average speeds calculated from tractor displacement were used instead of time-resolved velocity measurements, which is also documented in the corresponding metadata file.

### 4.5 Surface profile measurements

**Fig. 6:**
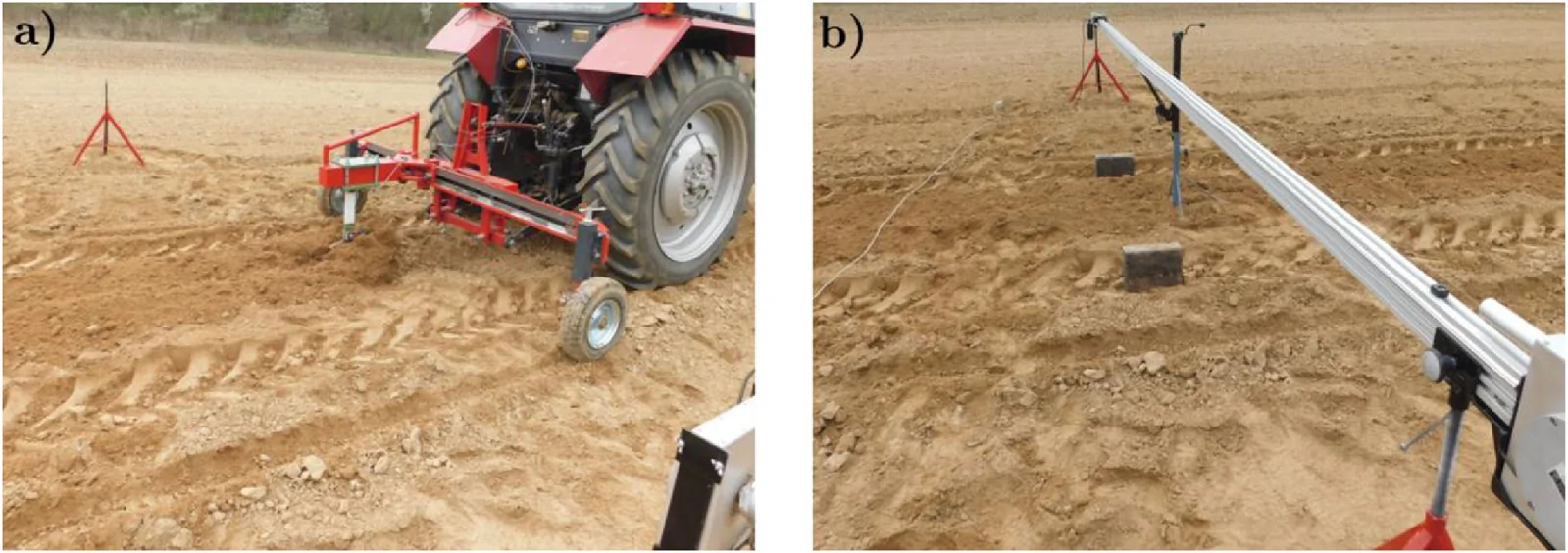
(a) The tractor equipped with the sweep tool passing the location of the surface profile measurement and leaving a burrow. (b) The laser profilograph used for surface profile measurement. Pictures taken during the measurement campaign carried out at medium moisture.

Surface profile measurements were performed to document changes in the soil profile caused by the passing sweep tool. Profiles were recorded immediately before and after each tillage run. The location of the surface profile measurement was reproducible as both ends of the profilograph were fixed, as depicted in Fig. 6a. The measurements were carried out using a laser profilograph consisting of an OPTRON ODS 750 laser sensor for surface elevation measurement (range: 400–1100 mm, resolution: 0.5 mm, serial number: 105–107, article number: 7007,753) and an ASM WS19KT-15,000 cable-extension position sensor for longitudinal position measurement (range: 15,000 mm, resolution: 0.146 mm, serial number: WS0947281498). The profilograph setup on the field is shown in Fig. 6b. The instrument was positioned perpendicular to the tillage direction and used to record the soil surface elevation across the tillage track. Following completion of the tillage run, the same procedure was repeated at the exact same location. The resulting datasets contain the measured surface elevation as a function of horizontal position before and after tillage. The recorded surface profiles were also post-processed to enable direct comparison between measurement runs. The horizontal coordinates were shifted so that the centerline of the tillage tool corresponded to *y* *=* *0.* Furthermore, the elevation values were shifted to obtain a zero mean elevation for the surface profile measured before tillage. The same elevation offset was applied to the corresponding post-tillage profile.

## Limitations

The main limitation of the presented dataset is the absence of surface profile measurements for the wet soil condition. Furthermore, the measurements were conducted in an agricultural field, where soil properties exhibit natural variability. Variations in soil moisture, compaction, and structure may therefore contribute to scatter in the cone penetration resistance, draft force, and surface profile measurements. Although this variability reflects realistic field conditions, it may increase the deviation of measured responses compared to laboratory experiments.

## Ethics Statement

The authors have read and follow the ethical requirements for publication in Data in Brief and confirming that the current work does not involve human subjects, animal experiments, or any data collected from social media platforms.

## CRediT authorship contribution statement

**Dániel Nagy:** Writing – original draft, Visualization, Validation, Software, Formal analysis, Data curation, Conceptualization. **Kornél Tamás:** Writing – review & editing, Investigation, Supervision, Resources, Project administration, Methodology, Funding acquisition, Formal analysis, Conceptualization.
